# Discrete Emotions and Depressive Symptoms in Family Dementia Caregivers

**DOI:** 10.1002/alz70861_108278

**Published:** 2025-12-23

**Authors:** Dustin Gad, Jenna L. Wells, Joan K. Monin

**Affiliations:** ^1^ Cornell University, Ithaca, NY USA; ^2^ Yale School of Public Health, New Haven, CT USA

## Abstract

**Background:**

Dementia family caregivers experience high rates of depression. Although greater sadness is commonly associated with depression, less is known about which specific emotions are linked with caregivers’ depression. Indeed, caregiving involves experiences that elicit a variety of negative emotions (e.g., fear about the progressive nature of the illness, shame regarding care recipients’ symptoms). Even less is known about the specific positive emotions that contribute to caregivers’ depression. Further, adult‐children and spousal caregivers have different responsibilities and pressures related to the caregiving role that may elicit unique emotions. Identifying the specific emotions underlying caregivers’ depression could lead to more targeted treatments.

**Method:**

We analyzed data from two independent samples. Study 1 (*N* = 145) consisted mainly of adult‐child caregivers from the northeast and Study 2 (*N* = 152) consisted mainly of spousal caregivers from the San Francisco Bay Area. Both samples completed self‐report measures of their emotions (PANAS) and depression (CES‐D).

**Result:**

Across both studies, all negative emotions were significantly correlated with greater depression and all positive emotions were significantly correlated with lower depression (*p*s < .011). For each study, we conducted two multiple regressions (one with PANAS negative emotions, and one with PANAS positive emotions, predicting depression). We then conducted a final multiple regression including the negative and positive emotions that were significant in these regressions. For Study 1, feeling greater distress (*B* = .301), upset (*B* = .169), shame (*B* = .139), fear (*B* = .246), and lower pride (*B* = ‐.119) remained associated with greater depression (*p*s < .047). For Study 2, feeling greater sadness (*B* = .379), distress (*B* = .269;), shame (*B* = .155), hostility (*B* = .186), and lower enthusiasm (*B* = ‐.200) remained associated with greater depression (*p*s < .003).

**Conclusion:**

Distress and shame were associated with greater depression for both adult‐children and spouses. Interventions that decrease distress and shame (e.g., addressing stigma, positively reappraising caregiving demands) may reduce depression across caregiving relationships. Lower depression was associated with greater pride in adult‐children and greater enthusiasm in spouses, suggesting that the specific positive emotions that buffer against depression may differ depending on relationship type.

